# Serum prolactin concentrations as risk factor of metabolic syndrome or type 2 diabetes?

**DOI:** 10.1186/1472-6823-13-12

**Published:** 2013-03-21

**Authors:** Lisa Balbach, Henri Wallaschofski, Henry Völzke, Matthias Nauck, Marcus Dörr, Robin Haring

**Affiliations:** 1 Institute of Clinical Chemistry and Laboratory Medicine, University Medicine Greifswald, Ferdinand-Sauerbruch-Straße, Greifswald, 17475, Germany; 2 DZHK (German Centre for Cardiovascular Research), partner site Greifswald, Greifswald, Germany; 3 Institute for Community Medicine, University Medicine Greifswald, Walther-Rathenau-Straße 48, Greifswald, 17475, Germany; 4 Department of Cardiology, University Medicine Greifswald, Ferdinand-Sauerbruch-Straße, Greifswald, 17475, Germany

## Abstract

**Background:**

To investigate potential associations of serum prolactin concentration (PRL) with metabolic syndrome (MetS) and type 2 diabetes mellitus (T2DM), previously observed in small and selected study samples, in a large population-based cohort.

**Methods:**

Data from 3,993 individuals (2,027 women) aged 20-79 years from the population-based Study of Health of Pomerania (SHIP) were used to analyse cross-sectional and longitudinal associations of PRL with MetS and T2DM risk in age- and multivariable-adjusted Poisson regression models. PRL were log-transformed and modelled as continuous (per standard deviation (SD) increase) and categorical predictor (sex-specific quartiles) variable, separately for men and woman.

**Results:**

Cross-sectional analyses showed an inverse association between low PRL concentrations and prevalent T2DM risk in men and women after multivariable-adjustment (men: Q1 vs. Q4: relative risk (RR), 1.55; 95% confidence interval (CI), 1.13 – 2.14; women: Q1 vs. Q4: RR, 1.70; 95% CI, 1.10 – 2.62). Likewise, higher PRL concentrations were associated with significantly lower T2DM risk (RR per SD increase in log-PRL: 0.83; 95% CI, 0.72 – 0.95 in men, and 0.84; 95% CI, 0.71 – 0.98 in women, respectively). An inverse association between PRL and MetS risk was not retained after multivariable adjustment. Longitudinal analyses yielded no association of PRL with incident MetS or T2DM.

**Conclusion:**

The present study is the first large population-based study reporting a cross-sectional inverse association between PRL and prevalent T2DM in both genders. But the absent longitudinal associations do not support a causal role of PRL as a risk factor of incident MetS or T2DM.

## Background

Prolactin (PRL) is a pituitary hormone essential for various physiological functions in the human body [1-3]. It is not only important for the initiation and maintenance of lactation, but seems to be also involved in reproduction, growth and development, osmoregulation, immune regulation, brain function, behaviour, and metabolism [1-3]. These different functions of PRL can only be fulfilled due to the fact that the PRL receptor is expressed in different tissues and cells such as lymphoid cells, endometrium, prostate, and adipocytes [1-3].

The metabolic syndrome (MetS), a cluster of cardiometabolic risk factors including obesity, hypertriglyceridemia, hypertension, and insulin resistance [4,5], is often associated with type 2 diabetes mellitus (T2DM) [1]. Although previous investigations about the potential effects of PRL in T2DM and its complications are scarce, existing experimental studies suggest an influence of PRL on T2DM via its metabolic effects on adipose tissue [2,3], development and growth of pancreatic ß-cells [6,7], insulin resistance [3,8], and lipid metabolism [3,9]. The ability of PRL to stimulate insulin [6] and suppress adiponectin as well as interleukin-6 release further suggests a potential role in the manifestation of insulin resistance [2]. But although these studies support the view that PRL promotes the growth and survival of pancreatic ß-cells and supports insulin secretion [6,7], other studies were not able to detect any correlation between PRL and metabolic disturbances [10].

An observational study among erectile dysfunction patients showed an association between low PRL concentrations and adverse cardiovascular risk profiles and events [11]. However, in most of these previous studies [10,11] the correlation between PRL and cardiometabolic risk factors including MetS or T2DM were not the main focus. Furthermore, previous studies were conducted in comparably small and selected patient populations without consideration of major confounding factors. Therefore, we aimed to investigate potential associations of PRL with MetS and T2DM using a large, representative sample of 3,993 individuals from the population-based longitudinal cohort Study of Health in Pomerania (SHIP).

## Methods

### Study population

The SHIP is a population-based cohort study in West Pomerania, a region in north-eastern Germany. Details regarding the design, recruitment, and procedures of the SHIP study have been published previously [12]. In brief, from the total population of West Pomerania comprising 213,057 inhabitants, a two-stage stratified cluster sample of adults aged 20-79 years was drawn in 1996. The net sample (without migrated or deceased persons) comprised 6,265 eligible subjects. Only individuals with German citizenship and main residency in the study area were included. All subjects received a maximum of three written invitations. In cases of non-response, letters were followed by repeated phone calls or by home visits if contact by phone was not possible [13]. After written informed consent was obtained, 4,308 (2,192 women) participants were examined (response 68.8%) between 1997 and 2001. During the five-year follow-up, 3,300 (1,711 women) participants were re-examined (response 83.6%) between 2002 and 2006. The study conformed to the principles of the Declaration of Helsinki and the Ethics Committee of the University of Greifswald approved the protocol. Out of 4,308 baseline participants, we excluded individuals with missing PRL data (N = 169), measured PRL >100.0 μg/l (N = 16), pregnancy (N = 8), pituitary disease (N = 1), and missing covariate data (N = 121), yielding a final study sample of 3,993 individuals (2,027 women).

### Measures

Information about socio-demographic characteristics and health-related lifestyle including smoking habits (categorized into current, former, and never-smokers), educational level (< 10, = 10, or > 10 years at school), parity (number of children), physical activity (< 1 h/week physical training during summer or winter) and alcohol consumption were collected by personal interviews. Waist circumference was measured to the nearest 0.1 cm using an inelastic tape midway between the lower rib margin and the iliac crest in the horizontal plane, with the subject standing comfortably with weight distributed evenly on both feet. Height and weight were measured for the calculation of body mass index (BMI) (weight [kg]/height [m]^2^). After a resting period of at least five minutes, systolic and diastolic blood pressure was measured three times on the right arm of seated participants with an oscillometric digital blood pressure monitor (HEM-705CP, Omron Corporation, Tokyo, Japan). The interval between the readings was three minutes. The mean systolic and diastolic blood pressure was calculated from the second and third measurement [14]. Menopausal status (pre- and post-menopausal) was defined according to a previously established definition from our cohort: pre-menopausal: all women < 40 years of age and between 40 – 60 years who reported menstrual cycle; post-menopausal: all women ≥ 60 years of age and all women between 40 – 60 years who reported no menstrual cycle [15].

Serum high-density lipoprotein (HDL) cholesterol concentrations were measured photometrically at baseline (Hitachi 704, Roche, Mannheim, Germany), whereas follow-up HDL concentrations were quantified by lipid electrophoresis (HELENA SAS-3 system, Helena 7 BioSciences Europe, Tyne & Wear, UK). To ensure comparability in the longitudinal HDL analyses, we used baseline HDL concentrations as the reference and calculated corrected follow-up HDL concentrations based on a previously published conversion formula [16]. Doing so, we found that the average HDL concentrations produced by the two methods were virtually identical, suggesting that the differences in HDL will be small within the range of practical relevance [17]. Serum triglycerides and glucose concentrations were determined enzymatically using reagents from Roche Diagnostics (Hitachi 717, Roche Diagnostics, Mannheim, Germany). Glycated hemoglobin (HbA1c) was determined by high-performance liquid chromatography (Bio-Rad, Munich, Germany). During the course of the study, the inter-assay coefficient of variation for HbA1c was 2.76% in the low pool and 1.38% in the high pool. All assays were performed according to the manufacturers’ recommendations by skilled technical personnel and internal quality controls were analyzed daily. In addition, the laboratory participates in official quarterly German external proficiency testing programs.

T2DM was defined based on self-reported physician’s diagnosis or use of antidiabetic medication (ATC code A10) in the last seven days, or HbA1c concentrations > 6.5%. Diagnostic criteria for the assessment of MetS components were defined according the Joint Scientific Statement to harmonize MetS [18] and modified for the use of non-fasting blood samples, as previously established in SHIP [19-22] and other large cohort studies [23]:

1. Elevated WC: men ≥ 94 cm; women ≥ 80 cm;

2. Elevated non-fasting glucose: ≥ 8.0 mmol/l or antidiabetic treatment (ATC codes A10A, A10B);

3. Decreased HDL cholesterol: men < 1.0 mmol/l, women < 1.3 mmol/l, or lipid-lowering treatment (ATC C10AB, A10AD);

4. Elevated non-fasting triglycerides: ≥ 2.3 mmol/l or lipid-lowering treatment (ATC C10AB, A10AD);

5. Elevated blood pressure: ≥ 130/85 mmHg or self-reported antihypertensive drug treatment. Participants fulfilling at least three out of these five components were assigned to MetS.

Serum PRL concentrations were measured from frozen sera of baseline participants. Non-fasting blood samples were drawn from the cubital vein in the supine position between 7.00 a.m. and 7.00 p.m. Samples were stored at -80°C until analysis using a chemiluminescent immunometric assay on an Immulite 2500 analyzer (Ref. L5KPR, DPC Biermann GmbH, Bad Nauheim, Germany). An aliquot of two alternating levels of a third party commercial control material (Bio-Rad Lyphochek Immunoassay Plus Control, lot 40151 and lot 40152; Bio-Rad, Munich, Germany) was included in each series in single determination. During the course of the study the inter-assay coefficient of variation was 5.6% with a systematic deviation of -4.3% at the 6.3 μg/l level, and 4.3% with a systematic deviation of -6.4% at the 14.5 μg/l level. The analytical sensitivity was 0.5 μg/l by an effective measurement range of 0.5 – 150 μg/l.

### Statistical analysis

Categorical data are expressed as percentage and continuous data as median (p25^th^, p75^th^). Sex-related differences were tested using the χ^2^ test (categorical data) and Mann-Whitney-U test (continuous data). We log-transformed the highly skewed PRL variable to achieve normality. To analyse cross-sectional and longitudinal associations of PRL with MetS and T2DM, we implemented age- and multivariable-adjusted Poisson regression models with robust standard errors and report the relative risks (RR) and their 95% confidence intervals (95% CI) per SD increase in log-PRL and for sex-specific PRL quartiles (reference: quartile four). Longitudinal incidence analyses were performed only in individuals without MetS and T2DM at baseline, respectively. P for trend test was conducted by including PRL quartiles as an ordinal score into the regression models. Covariates adjusted for in multivariable regression models included age, BMI, smoking status, physical activity, educational level, and alcohol consumption. All analyses were performed separately in men and women.

Several sensitivity analyses were performed. In women, we additionally adjust multivariable regression models for menopausal status and parity. On the basis of ATC codes for metoclopramide (A03FA01, A03FA03, N02CX59), cimetidine (A02BA01), reserpine (C02LA01), methyldopa (C02AB), and psychoanaleptics (ATC-code N06), we excluded 184 individuals indicative of these PRL influencing medications [24] and repeated the multivariable regression models accordingly. Furthermore, we used inverse probability weights to adjusted our longitudinal analyses for possible non-response bias from drop-out between the baseline and follow-up examination [25]. Finally, we additionally adjusted the multivariable models for blood sampling time to evaluate the potential impact of the diurnal cycle in PRL secretion on the revealed estimates. Two-sided probability values < 0.05 were considered statistically significant. All the statistical analyses were performed using Stata 11.0 (Stata Corporation, College Station, TX, USA).

## Results

Comparing the baseline characteristics of the study sample by sex, men showed significantly lower PRL concentrations and a higher cardiometabolic risk factor level (with regard to smoking, alcohol consumption, BMI, blood pressure, lipid levels and parameters of glycemic control) than women (Table 1). Also the prevalence of MetS (34.0% vs. 22.2%) and T2DM (12.6% vs. 9.0%) was higher in men compared to women.

**Table 1:** Baseline characteristics of the study population stratified by sex

	**Men**	**Women**	p-value*
	**(N = 1,966)**	**(N = 2,027)**	
Age, years	52.1 (37.4; 65.3)	49.5 (36.0; 62.3)	<0.001
Serum prolactin, μg/l	4.9 (3.6; 6.9)	6.5 (4.5; 9.4)	<0.001
Current smoker,%	33.5	26.9	<0.001
Physical activity,%	41.0	43.4	0.130
Alcohol consumption, g/d	11.9 (1.5; 27.9)	2.5 (0.0; 7.2)	<0.001
Educational level			<0.001
< 10 years	42.7	37.8	
= 10 years	39.8	46.8	
> 10 years	17.5	15.3	
Parity, N	NA	2 (1, 3)	NA
Body mass index, kg/m^2^	27.3 (24.9; 29.9)	26.1 (22.8; 30.2)	<0.001
Waist circumference, cm	95.2 (87.5; 102.9)	81.5 (72.8; 92.1)	<0.001
Systolic blood pressure, mmHg	140.5 (129.5; 153.0)	127.0 (114.5; 143.0)	<0.001
Diastolic blood pressure, mmHg	85.0 (78.0; 93.0)	80.5 (73.5; 87.5)	<0.001
Serum total cholesterol, mmol/l	5.7 (4.9; 6.4)	5.7 (4.9; 6.5)	0.811
Serum triglycerides, mmol/l	1.7 (1.2; 2.6)	1.3 (0.9; 1.9)	<0.001
Serum LDL cholesterol, mmol/l	3.6 (2.8; 4.3)	3.4 (2.7; 4.2)	0.002
Serum HDL cholesterol, mmol/l	1.2 (1.0; 1.5)	1.5 (1.3; 1.8)	<0.001
Serum glucose, mmol/l	5.4 (5.0; 6.0)	5.2 (4.8; 5.7)	<0.001
Glycated hemoglobin A1c,%	5.4 (5.0; 5.9)	5.2 (4.8; 5.7)	<0.001
Metabolic syndrome,%	34.0	22.2	<0.001
Type 2 diabetes mellitus,%	12.6	9.0	<0.001

Data are given as percentages or median (p25^th^; p75^th^). NA, not applicable; LDL cholesterol, low-density lipoprotein cholesterol; HDL cholesterol, high-density lipoprotein cholesterol.

* Differences between men and women were tested using χ^2^ test (categorical data) and Whitney-Mann-U Test (continuous data).

Cross-sectional age-adjusted regression models showed an inverse association between PRL and MetS risk in women (Q1 vs. Q4: RR, 1.32; 95% CI, 1.04 – 1.66), but not in men (Q1 vs. Q4: RR, 1.15; 95% CI, 0.97 – 1.37). This sex-specific association was not retained after multivariable adjustment (Table 2). Low PRL concentrations were consistently associated with T2DM in age- and multivariable-adjusted regression models (men: Q1 vs. Q4: RR, 1.55; 95% CI, 1.13 – 2.14; women: Q1 vs. Q4: RR, 1.70; 95% CI, 1.10 – 2.62). Furthermore, p for trend analyses showed a progressive inverse relationship across PRL quartiles. Likewise, higher PRL concentrations were associated with a significantly lower T2DM risk (RR per SD increase in log-PRL: 0.83; 95% CI, 0.72 – 0.95 in men, and 0.84; 95% CI, 0.71 – 0.98 in women, respectively).

**Table 2:** Cross-sectional and longitudinal associations of serum prolactin with metabolic syndrome and type 2 diabetes mellitus

	**Metabolic syndrome**				**Type 2 diabetes mellitus**			
	**Cross-sectional**		**Longitudinal**		**Cross-sectional**		**Longitudinal**	
	**Age-adjusted model**	**Multivariable model**	**Age-adjusted model**	**Multivariable model**	**Age-adjusted model**	**Multivariable model**	**Age-adjusted model**	**Multivariable model**
**Prolactin**	**Men**							
**per SD increase**	0.95 (0.89; 1.02)	0.95 (0.89; 1.01)	1.05 (0.95; 1.15)	1.04 (0.95; 1.14)	0.84 (0.73; 0.96)*	0.83 (0.72; 0.95)*	1.20 (0.98; 1.47)	1.19 (0.96; 1.47)
**Quartiles (Q4 Ref.)**								
**Q1**	1.15 (0.97; 1.37)	1.15 (0.98; 1.35)	0.83 (0.64; 1.06)	0.87 (0.68; 1.10)	1.55 (1.12; 2.13)*	1.55 (1.13; 2.14)*	0.71 (0.41; 1.23)	0.78 (0.45; 1.35)
**Q2**	1.06 (0.89; 1.28)	1.03 (0.87; 1.22)	0.94 (0.74; 1.19)	0.94 (0.75; 1.18)	1.14 (0.81; 1.61)	1.11 (0.79; 1.56)	0.73 (0.42; 1.26)	0.78 (0.45; 1.33)
**Q3**	1.04 (0.87; 1.25)	1.02 (0.85; 1.21)	0.91 (0.72; 1.16)	0.94 (0.75; 1.18)	0.92 (0.63; 1.33)	0.92 (0.64; 1.33)	0.76 (0.44; 1.32)	0.72 (0.41; 1.27)
**P for Trend**	0.104	0.076	0.172	0.276	0.002	0.002	0.253	0.472
	**Women**							
**per SD increase**	0.92 (0.85; 1.00)	0.97 (0.90; 1.05)	0.89 (0.79; 1.00)	0.92 (0.83; 1.02)	0.81 (0.68; 0.95)*	0.84 (0.71; 0.98)*	1.09 (0.82; 1.45)	1.13 (0.89; 1.44)
**Quartiles (Q4 Ref.)**								
**Q1**	1.32 (1.04; 1.66)*	1.11 (0.89; 1.39)	1.26 (0.95; 1.68)	1.15 (0.86; 1.53)	1.89 (1.21; 2.96)*	1.70 (1.10; 2.62)*	0.88 (0.44; 1.78)	0.73 (0.37; 1.43)
**Q2**	1.13 (0.89; 1.44)	1.04 (0.83; 1.32)	1.22 (0.91; 1.63)	1.16 (0.87; 1.54)	1.82 (1.15; 2.86)*	1.69 (1.09; 2.63)*	0.81 (0.39; 1.68)	0.75 (0.37; 1.50)
**Q3**	1.11 (0.86; 1.44)	1.07 (0.83; 1.32)	0.73 (0.51; 1.04)	0.78 (0.55; 1.11)	1.29 (0.77; 2.16)	1.26 (0.76; 2.09)	1.00 (0.48; 2.06)	1.08 (0.53; 2.20)
**P for Trend**	0.016	0.399	0.013	0.106	0.001	0.007	0.633	0.224

* p < 0.05; SD, standard deviation. The multivariable model was adjusted for age, body mass index, smoking status (three categories), physical activity, educational level (three categories), and alcohol consumption.

**Figure 1:**
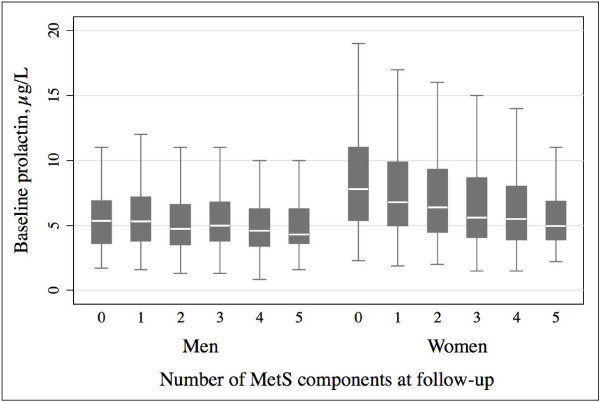
**Boxplots for median baseline prolactin concentrations (25**^**th **^**and 75**^**th **^**percentile) according to the follow-up number of metabolic syndrome (MetS) components, separately for men and women.** One-way analysis of variance showed a significant inverse trend for prolactin concentrations across number of MetS components at follow-up in women (p = 0.033), but not in men (p = 0.331).

After a median follow-up time of 5.0 years (range 4.4 – 8.5 years) 3,078 individuals (1,589 women) were repeatedly examined. Among the individuals without the respective condition at baseline, 27.7% developed incident MetS and 5.3% incident T2DM. Longitudinal analyses revealed a significant trend of lower PRL concentrations with increasing number of MetS components in women (p = 0.033), but not in men (p = 0.331) (Figure 1). Multivariable regression models showed no association of PRL with incident MetS and T2DM (Table 2). The conducted sensitivity analyses with 1) additional adjustment for menopausal status and parity, 2) exclusion of individuals indicative of PRL influencing medications, 3) additional inclusion of ‘drop-out’ weights and 4) additional adjustment for blood sampling time showed no impact on the overall estimates (data not shown).

## Discussion

To our knowledge, this is the first population-based study to show a cross-sectional inverse association between PRL and T2DM risk in both men and women. Using a large sample of 3,993 individuals from the general population, age- and multivariable-adjusted regression models detected an inverse association between low PRL concentrations and increased risk for prevalent T2DM.

These findings are in line with previous observational studies [11,26,27] of the relationship between PRL and T2DM. Among those, the only large-scaled study was conducted in 2,351 male patients with sexual dysfunction, in whom low PRL concentrations were associated with an increased number of MetS factors [26]. Similarly to ours, that study [26] and its subsequent follow-up [11] showed an inverse association between serum PRL concentrations and prevalent T2DM. However, since both studies based on male patient data, the comparability with the present population-based findings is quite limited. Furthermore, a cross-sectional study in 345 healthy volunteers aged 30-55 years showed a negative association of PRL with insulin resistance and a doubled risk of prevalent MetS per SD decrease in PRL [27].

Evidence from cross-sectional patient-based studies in drug naive schizophrenia patients suggests that high dopamine and subsequently low PRL concentrations [28,29] are related to an increased T2DM risk, including insulin resistance and impaired fasting glucose [30]. Thus, one may speculate if common anti-psychotics could probably protect from T2DM onset and progression by increasing PRL concentrations through the inhibition of the D2 receptor [31]. However, since previous studies [28-30] were exclusively cross-sectional, cause and effect in the association of PRL with T2DM are difficult to determine. For example, a small interventional study in turn suggests insulin secretion as the major determinant of PRL release [9].

In the search of potential explanations, two previous studies related alterations of serotonergic pathways caused by low PRL concentrations to the pathogenesis of MetS [27,32]. Furthermore, PRL-receptor deficient mice show an impaired development of pancreatic ß-cells, finally leading to a blunted insulin response and mild glucose intolerance [7]. In contrast to our findings, studies including pathological hyperprolactinemia caused by PRL-secreting tumors (prolactinomas) showed that, after treatment with dopamine agonists, reduced PRL concentrations led to increased insulin sensitivity [33,34].

These results, however, were collected in small patient-based samples with clinically confirmed prolactinoma and serum PRL concentrations outside the reference range, thus limiting its transferability to the general population. On contrary, HOMA-IR, as a measure of insulin resistance, did not change after treatment with dopamine agonists [33], and PRL concentrations did not correlate with HOMA-IR change or decrease in glucose, respectively [8,34]. Interestingly, a previous cross-sectional study in obese non-prolactinoma patients showed similar absent correlations of PRL with insulin, HOMA-IR, and glucose levels [10]. Available in-vitro studies rather suggest an influence of prolactin on ß-cell secretion via increased glucokinase activity [35], improved ß-cell specific survival [36,37], or inhibition of intrinsic ß-cell apoptosis [38]. However, to provide the necessary level of detail to elucidate the exact mechanisms of PRL on different tissues and its impact on cardiometabolic risk factors further experimental in-vitro research is needed.

We previously published findings from our study suggesting a positive association of PRL with biomarkers of inflammation [39] and mortality (cardiovascular and all-cause mortality) [40], as well as an inverse association of PRL with cardiac remodelling [41]. However, these observational findings from our and other studies do not provide consistent risk associations, suggesting the need for further investigations into the potential role of PRL as cardiometabolic risk marker.

For the interpretation of the present study, the following strengths and limitations need to be considered. Major strengths include the large sample, the longitudinal study design, and a broad age range. Furthermore, comprehensive sensitivity analyses were conducted to assess the validity of our findings. An important limitation of this study includes the reliance on single serum PRL measurements based on non-fasting blood samples, which is not adapted to the pulsatile release of PRL. But it was shown that the pulsatile secretion occurs mostly during the night and is relatively constant between 9.00 a.m. and 5.00 p.m. [42]. Further limitations arise from the Caucasian study sample, which restricts the generalizability of our results. Finally, we did not exclude other endocrine diseases, which possibly trigger T2DM including hyperthyroidism [43,44], acromegaly and the Cushing Syndrome [45].

However, given the inherent limitations of observational research [46], the definite role of serum PRL concentrations as a risk factor or risk marker can not be elucidated based on epidemiological data alone. Previous associations between serum PRL concentrations and disease risk observed in clinical study samples, but not in the general population, suggest PRL rather as specific disease marker than as causal risk factor for MetS and T2DM. Thus, its application and significance in daily clinical practice is very limited to date.

## Conclusions

In summary, this is the first population-based study to show low PRL concentrations related to a higher T2DM risk in both genders. But given the variable associations between PRL and T2DM, but not with MetS, together with the absent longitudinal associations with both outcomes, the present study does not support PRL as a causal cardiometabolic risk factor. Therefore, we hypothesize PRL rather as a marker of the complex multi-level alterations involved in the onset and progression of T2DM. However, to further elucidate the potential role of PRL in the context of this complex interplay, further observational as well as interventional research is needed.

## Competing interest

There is no conflict of interest that could be perceived as prejudicing the impartiality of the research reported.

## Authors’ contributions

RH, LB, and HW contributed to the study design and ideas for the data analysis. HV, MD, MN, and HW organized the sample collection and data preparation. Statistical analyses were performed by RH. LB, RH, and HW contributed to the interpretation of the results and the discussion. LH drafted the manuscript and wrote the final version together with all other co-authors. All authors read, critically revised, and finally gave approval of the version to be published.

## Pre-publication history

The pre-publication history for this paper can be accessed here:

http://www.biomedcentral.com/1472-6823/13/12/prepub
